# Protective and risk factors in amateur equestrians and description of injury patterns: A retrospective data analysis and a case - control survey

**DOI:** 10.1186/1752-2897-5-4

**Published:** 2011-02-04

**Authors:** Rebecca M Hasler, Lena Gyssler, Lorin Benneker, Luca Martinolli, Andreas Schötzau, Heinz Zimmermann, Aristomenis K Exadaktylos

**Affiliations:** 1 University of Bern, Department of Emergency Medicine, Inselspital, Bern, Switzerland; 2 University of Bern, Department of Orthopaedic Surgery, Inselspital, Bern, Switzerland; 3 Schötzau und Simmen, Statistical Consulting, Basel, Switzerland

## Abstract

**Background:**

In Switzerland there are about 150,000 equestrians. Horse related injuries, including head and spinal injuries, are frequently treated at our level I trauma centre.

**Objectives:**

To analyse injury patterns, protective factors, and risk factors related to horse riding, and to define groups of safer riders and those at greater risk

**Methods:**

We present a retrospective and a case-control survey at conducted a tertiary trauma centre in Bern, Switzerland.

Injured equestrians from July 2000 - June 2006 were retrospectively classified by injury pattern and neurological symptoms. Injured equestrians from July-December 2008 were prospectively collected using a questionnaire with 17 variables. The same questionnaire was applied in non-injured controls. Multiple logistic regression was performed, and combined risk factors were calculated using inference trees.

**Results:**

**Retrospective survey**

A total of 528 injuries occured in 365 patients. The injury pattern revealed as follows: extremities (32%: upper 17%, lower 15%), head (24%), spine (14%), thorax (9%), face (9%), pelvis (7%) and abdomen (2%). Two injuries were fatal. One case resulted in quadriplegia, one in paraplegia.

**Case-control survey**

61 patients and 102 controls (patients: 72% female, 28% male; controls: 63% female, 37% male) were included. Falls were most frequent (65%), followed by horse kicks (19%) and horse bites (2%). Variables statistically significant for the controls were: Older age (p = 0.015), male gender (p = 0.04) and holding a diploma in horse riding (p = 0.004). Inference trees revealed typical groups less and more likely to suffer injury.

**Conclusions:**

Experience with riding and having passed a diploma in horse riding seem to be protective factors. Educational levels and injury risk should be graded within an educational level-injury risk index.

## Background

The role of horses in society has completely changed. Once considered as working animals and as a means of transport animals, horses are nowadays used primarily for leisure and sports activities, at least in the Western World [1-3].

Today, in Switzerland there are an estimated 150,000 equestrians (2% in a population of 7.2 million), and more than 65,000 belong to equestrian and country clubs - about 3-4 times more than only 7 years ago (unpublished data, Swiss Equestrian Association) [1]. These numbers are comparable with other western European countries. A qualification in horse riding is not a standard in Switzerland. Since 1990, 70,000 equestrians hold a diploma and 8,500 a licence in horse riding[Unpublished information of the Swiss Equestrian Association]. Switzerland is a member of the Fédération Equestre International and follows their rules and standards [4]. The new emphasis on sports has been accompanied by typical riding injuries. Horse riding is considered more dangerous than motorcycle riding, skiing, automobile racing, football and rugby [5,6]. Some reports of injuries among non-professional equestrians, jockeys, polo players, rodeo riders and cross country riders have been published in the past decades [7-13]. Although horse riding injuries have decreased by about 40% over the past 20 years - probably because of improved safety guidelines for riders - they still result in a significant number of injuries, including head and spinal injuries, which may be associated with global functional impairment and give rise to long-term disability [14-16]. Loder et al. detected more 5,033 equestrian related injuries in the US national trauma database between 2002 and 2004. Spinal injuries accounted for 2.4% of all injuries and head/neck trauma for 23.8% [14].

Among several regional hospitals caring for injured equestrians in Switzerland, we are the only level I trauma center in this region providing spinal, neurosurgery, thoracic, hand or plastic surgery. The large number of riding injuries at our trauma center and the rising costs of healthcare prompted our institution to evaluate injury patterns as well as to look for ways of more effectively preventing injuries. The study goals were to (1) analyze injury patterns and mechanisms, (2) to establish general protective and risk factors in horse riding and (3) to define groups of equestrians at high risk.

## Methods

### Setting

Our Emergency Department is the only level I accident and emergency unit in its catchment area of about 1.8 million people and treats about 15,000 trauma cases per year, 450 of them with an injury severity score >12.

### Ethical considerations

Participation in the study was voluntary and anonymous, and confidentiality was guaranteed. The data were collected, stored, analyzed and shared according to the ethics committee standards of our institution.

### Retrospective survey

For the retrospective survey, all patients of at least 16 years of age admitted to our trauma center from 1 July 2000, through 30 June 2006 were reviewed in our computerized database (Qualicare Office, Medical Database Software, Qualidoc AG, Bern, Switzerland). Since that medical database allows instantaneous retrieval of past diagnostic reports, discharge summaries, other text documents, patients' laboratory results, or radiographs, the authors were able to retrospectively analyze the diagnostic results, and therapeutic procedures. We included all patients with acute traumatic injuries related to horse riding, including mounting and handling of the horse. Patients with chronic health issues related to horse riding were excluded, as well as patients with incomplete data. Furthermore, we excluded cases, which involved horses in other ways than horse riding itself (e.g. horse carriage drivers). 365 patients with horse riding related injuries (277 female, 88 male) were included. A total of 38 cases had to be excluded due to incomplete data or due to injuries involving horses, but not horse riding.

All patients were classified by age, gender, date of injury, 8 different types of injury (head, face, spine, upper extremities, lower extremities, thorax, pelvis and abdomen), mechanism of injury (fall, horse bite, horse kick and other) and persistent neurological symptoms. Injuries were classified using the AIS 2008 guidelines [17]. For head injuries the GCS was evaluated, additionally.

### Case-control survey

The patient group included 61 injured equestrians of 16 years or older admitted to our trauma center. The control group included 102 non-injured equestrians of 16 years or older and all levels of horse riding experience were included. Participants were interviewed by an instructed medical student using a questionnaire incorporating 17 potential risk factors (patients) or while horse riding at different places (controls) using the same questionnaire. Patients were asked to answer the questions concerning their ride when they suffered the injury or their last ride (controls).

We defined 17 unvalidated primary outcome measures as possible risk factors. Part of them has been used in prior risk assessment studies [18,19]. The risk factors included: Equestrian characteristics (age, gender, diploma in horse riding, horse riding proficiency, former riding injuries, readiness to take risks and ride at speed, abstinence from alcohol while horse riding, style of horse riding, riding own horse or another person's horse, riding alone, with a friend or with a group, horse riding hours per week, horse riding only for leisure or also competitively), use of protective equipment and horse characteristics (age, gender and breed of horse).

In the patient group, injury characteristics such as mechanism of the injury, scene of the injury, and presumed reason for the injury were also analyzed.

We included injuries in horse riding, including handling and mounting of the horse.

Patients with intracranial bleeding, skull fractures, Glasgow Coma Score <14 or persistent retrograde amnesia were excluded. Patients with concussions were included as long as their GCS was 15 and they were able to fully and coherently understand and answer the questions. Controls were eligible if they were uninjured equestrians and at least 16 years old.

The questionnaire was distributed to all patients attending the emergency department and fulfilling the inclusion criteria. It was furthermore distributed to uninjured controls at riding centres in the catchment area of the hospital. These riding centres are commercially led stables, where equestrians "park and ride" their horses. There was no incentive for participating in the study.

A total of 5 (7%) patients and 8 (7%) controls refused to participate due to a lack of time and/or no interest in participating in a study. We excluded 2 (3%) patients and 2 (2%) controls because of incomplete information.

The participants were divided into three age groups: (a) 16-30 years, (b) 31-45 years or (c) >45 years.

The number of hours riding per week were classified as (a) <10 h, (b) 10-19 h, (c) 20-29 h, (d) 30-39 h or (e) >39 h.

The degree of equestrian proficiency (assessed by the individual equestrian) was divided into (a) beginners, who were in their first year of practicing horse riding, (b) pre-intermediates, who had more than one year of riding experience, but practice horse riding for leisure purposes, (c) intermediates, who have more than one year of horse riding experience and take part in competitions regularly or (d) professionals, who perform horse riding as a profession.

Participants were also asked whether they had (a) a first diploma or (b) more than one diploma of the Swiss horse riding association. Applicants for the first diploma, called "brevet", do not have to fulfil an age limit. The exam consists of a theoretical part about handling of the horse and a practical course involving competencies like basic knowledge (mounting a horse, riding in gallop or over small obstacles), gaited horse riding, western riding or trail riding. The second diploma, called a "licence", consists of a three part exam and can be taken in different sub disciplines like jumping, dressage or endurance riding [3].

We included four different types of protective equipment: (a) helmets, (b) riding boots (c) gloves and (d) protective waistcoats.

Styles of horse riding included (a) dressage, (b) jumping, (c) eventing, (d) Western or (e) distance riding.

Western riding is a style of horseback riding which evolved from the ranching and warfare traditions brought to the Americas by the Spanish Conquistadors, and both equipment and riding style evolved to meet the working needs of the cowboy in the American West. Because of the necessity to control the horse with one hand and use a lasso with the other, western horses were trained to change direction with light pressure of a rein against the horse's neck. Thus a riding style developed that emphasized a deep, secure seat, and training methods encouraged a horse to be responsive on very light rein contact.

The readiness to take risks and ride at speed were measured on a self-reported visual analogue scale (VASrisk, VASspeed) from 0 to 10 (0 minimum).

The horse age groups were: (a) <5 years, (b) 5-14 years or (c) >14 years.

The horses were classified as (a) mare, (b) stallion or (c) gelding.

The type of horses was divided into (a) warm blooded, (b) cold blooded or (c) thoroughbred.

The following riding terrains were documented: (a) paved roads, (b) forest/field, (c) barn, (d) indoor riding hall, (e) paddock or (g) other places.

The presumed reason for injury was documented as: (a) horse frightened, (b) nervous disposition of horse, (c) refusal to jump, (d) slipping/stumbling of horse, (e) too close to another horse, (f) rider lost balance, (g) bucking, or (h) rider distracted or not paying attention. (Appendix 1)

### Statistical analysis

Only cases with complete data were analysed. To predict injury, single logistic regression was used to estimate the influence of all factors. On the basis of the number of patients and controls in our survey, we decided to perform multiple logistic regression for five selected factors: age, gender, helmet, diploma in horse riding and readiness for risk. As a rule the numbers of predictors should be smaller than the number of cases divided by ten. This prevents the model from over fitting [20]. Odds ratios (OR) and 95% confidence intervals (CI) were calculated.

The level of significance was p < 0.05. All calculations were done with R version 2.7.0 (A Language and Environment for Statistical Computing) [21,22]. For continuous and ordinal variables (scores), the OR has to be based on a meaningful difference for the predicting variable. These were expressed as the ratio of the odds from the 3rd to the 1st quartile based on cases and controls, representing a typical above-average to a typical below-average value.

Conditional inference tree analysis was applied to elicit possible combinations of two or more of the selected possible risk factors. A tree-based model is a good exploratory tool for the approximation of a complex model. The advantages are clearness of interpretation and visualization of complex interactions which are not covered by regression modelling. The disadvantage is possible instability because of the use of strongly correlated predictors and the splitting of continuous variables into classes [23,24].

## Results

### Retrospective survey

#### Demographics

Between 1 July 2000 and 30 June 2006 365 patients (100%) were included. Among those 76% (n = 277) were female. The mean ages were 30 years for women (16-76 years) and 41 years for men (16-78 years). The group of young women 16-30 years represented the largest group of the injured (40% of overall sample, n = 146).

#### Injury patterns

We observed 528 injuries (100%). Injuries to the extremities (32%, n = 173) (upper extremities: 17%, n = 92; and lower extremities: 15%, n = 81) were most frequent, followed by head injuries (24%, n = 127) and spinal injuries (14%, n = 76). Thoracic and facial injuries accounted for 9% (n = 50) each, pelvic injuries for 7% (n = 39) and abdominal injuries for 2% (n = 13). Most head injuries were mild with a GCS 15-13 and an AIS_head _<3 (60%, n = 76).

The median ISS was 9 (mean 12.0), with 93 patients (25.5%) having an ISS >16.

The injury pattern of patients with an AIS3+ is shown in table 1.

**Table 1:** Patients with injuries AIS3+ for each body region

	Patients (n)*	Patients (%)*
Head	51	14.0

Face	15	4.1

Chest	27	7.4

Abdomen	6	1.6

Spine	18	4.9

Upper limbs	5	1.4

Lower limbs	23	6.3

*with injury in that body region.

One patient (<1%) with severe head injury and one patient (<1%) with thoracic trauma and pericardial tamponade died. One injury (<1%) resulted in quadriplegia and another case (<1%) in paraplegia.

### Case-control survey

#### Demographics

Between 1 July 2008 and 31 December 2008, 61 patients (90%) and 102 (91%) non-injured controls were interviewed. The mean age of the patients was 42 years (range 18-81 years), and 35 years (range 17-70 years) for the controls. Women accounted for 72% (n = 44) of the patients and 63% (n = 64) of the controls. The mean experience in horse riding was 4 years (range for patients and controls: 0-5 years) for both the patients and controls.

#### Mechanisms of injury

Most frequent were falls from the horse (65%, n = 40), followed by kicks (19%, n = 12) and bites (2%, n = 1). In 14% (n = 8) of cases other mechanisms such as pulling too sharply or strongly on the reins or injuries while mounting the horse were responsible. The majority of the controls (80%, n = 82) and of the patients (83%, n = 51) had already sustained former injuries in horse riding.

#### Scene of the injury

Injuries were sustained on paved roads (29%, n = 18), in the forest or out on the field (26%, n = 16), in barns (16%, n = 10), in indoor riding halls (15%, n = 9) or in paddocks (16%, n = 10).

#### Presumed reason for injury

As the cause of the injury, 33% (n = 20) reported frightening of their horse, 8% (n = 5) attributed the injury to the nervous disposition of the horse, and 15% (n = 9) to a refusal to jump. The horse slipped or stumbled in 13% (n = 8) of cases, and had come too close to another horse in 7% (n = 4). The rider lost balance in 6% (n = 4) of cases, the horse bucked in 13% (n = 8), and distraction of the rider was given as the presumed reason of in 5% (n = 3) of cases.

#### Single logistic regression analysis

The following variables were statistically significant for the control group:

None of the other variables tested showed statistically significant differences:

As shown in tables 2 and 3, patients were older than the controls. The controls were more often out with a friend, whilst patients were more often riding alone or in a group. Horse riding between 20 and 29 hours per week was a significant result for controls as well as riding with one's own horse. More controls than patients wore helmets and protective waistcoats. More controls were involved in jumping. Controls had a higher level of equestrian proficiency and more often held one or more diplomas in horse riding. The patients had been riding with younger horses than the controls.

**Table 2:** Variables statistically significant for the control group

VARIABLES	OR	CI 95%	P-VALUE
**Equestrian characteristics**			

Age group: >45 years vs 16-30 years	3.7	1.26 - 10.88	0.018

Riding with a friend vs riding alone	0.41	0.21 - 0.78	0.007

Horse riding hours per week: 20 h - 29 h vs <10 h	0.37	0.15 - .0.93	0.034

Riding own horse vs another person`s horse	2.31	1.13 - 4.73	0.022

**Use of protective equipment**			

Use of helmet vs no use of helmet	0.35	0.17 - 0.74	0.006

Use of protective waistcoat vs no use of protective waistcoat	0.26	0.08 - 0.78	0.017

**Style of horse riding**			

Jumping vs other horse riding styles	0.28	0.14 - 0.55	<0.001

**Educational level**			

Level of horse riding: pre-intermediate vs beginner	0.19	0.04 - 0.87	0.032

Level of horse riding: intermediate vs pre-intermediate	0.23	0.06 - 0.83	0.025

Level of horse riding: professional vs intermediate	0.26	0.07 - 0.93	0.038

First diploma (brevet) in horse riding vs no diploma in horse riding	0.30	0.13 - 0.67	0.003

More than one diploma (brevet and licence) in horse riding vs no diploma in horse riding	0.23	0.10 - 0.53	<0.001

**Horse characteristics**			

Age of horse 5 - 14 years vs age of horse <5 years	0.19	0.06 - 0.57	0.003

Age of horse >15 years vs age of horse 5 - 14 years	0.14	0.04 - 0.47	0.002

**Table 3:** Variables statistically not significant

VARIABLES	OR	CI 95%	P-VALUE
**Equestrian characteristics**			

Age group: 31-45 years vs. 16-30 years	1.29	0.58 - 2.88	0.526

Age group: 31-45 years vs. 16-30 years	1.08	0.45 - 2.6	0.866

Gender of equestrian: female vs. male	1.54	0.77 - 3.06	0.221

Riding alone vs. not riding alone	0.99	0.51 - 1.94	0.988

Riding in a group vs. not riding in a group	0.78	0.38 - 1.58	0.486

Horse riding hours per week: 10 h - 19 h vs. <10 h	0.51	0.24 - 1.11	0.089

Horse riding hours per week: 30 h - 39 h vs. <10 h	1.11	0.25 - 4.88	0.889

Horse riding hours per week: >39 h vs. <10 h	1.11	0.25 - 4.88	0.889

Horse riding only for leisure vs. horse riding for leisure and competitions	0.96	0.47 - 1.98	0.917

Horse riding for leisure and competitions vs. horse riding only for leisure	0.61	0.32 - 1.16	0.128

Former accidents in horse riding vs. no former accidents in horse riding	0.98	0.46 - 2.08	0.609

**Use of protective equipment**			

Use of gloves vs. no use of gloves	1.13	0.56 - 2.28	0.732

Use of riding boots vs. no use of riding boots	2.85	0.60 - 13.67	0.189

**Style of horse riding**			

Dressage vs. other than dressage	0.57	0.28 - 1.16	0.123

Western vs. other than western	1.43	0.42 - 4.90	0.570

Distance vs. other than distance	0.41	0.04 - 3.74	0.428

Abstinence from alcohol vs. alcohol consumption	0.89	0.40 - 1.96	0.773

High readiness for speed vs. low readiness for speed	1.01	0.87 - 1.18	0.860

High readiness for risk vs. low readiness for risk	0.88	0.76 - 1.02	0.088

**Horse characteristics**			

Gender of horse: mare vs. stallion	0.43	0.08 - 2.30	0.326

Gender of horse: mare vs. gelding	0.66	0.34 - 1.27	0.215

Type of horse: warm blooded vs. cold blooded	0.45	0.05 - 4.12	0.478

Type of horse: warm blooded vs. thoroughbred	1.79	0.75 - 4.30	0.191

#### Multiple logistic regression analysis of effects of age, gender, use of helmet, diploma in horse riding and readiness for risk

**Figure 1:**
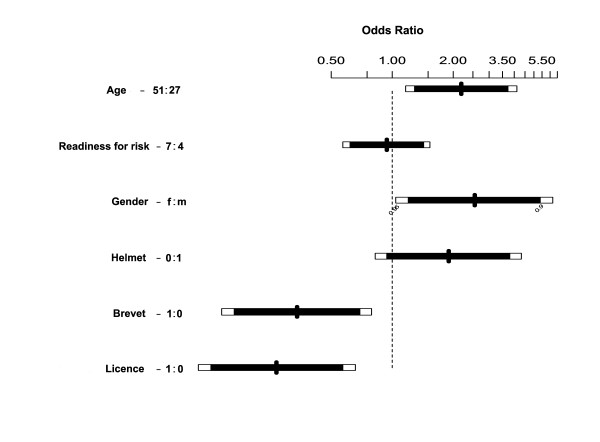
**Odds ratios of multiple logistic regression analysis for each variable**. The white part of the bar indicates 95% CI or more. Continuous (age) and ordinal variables (risk), are expressed as the ratio of the odds from the 3rd to the 1st quartile (listed in the numbers after the corresponding variable). Dichotomous variables are coded as follows: Gender (0 = male, 1 = female), helmet (1 = use of helmet, no use of helmet).

Statistically significant relationships between injury and the following factors were found (Figure 1):

- age (OR 1.03, 95% CI 1.01-1.06; p = 0.015)

- gender (OR 2.54, 95% CI 1.04-6.21; p = 0.04)

- having a diploma in horse riding (OR 0.27, 95% CI 0.11-0.65; p = 0.004)

Wearing a helmet was linked to a 50% risk reduction (OR 0.53, 95% CI 0.23 - 1.22; p = 0.134).

#### Conditional inference trees

**Figure 2:**
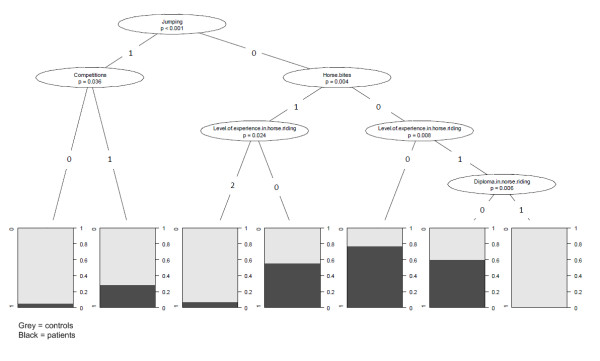
**Example of a conditional inference tree used to indicate groups of riders at less risk of injury and groups at risk by a combination of the most significant risk factors (see reference (23) for an explanation of the interpretation of conditional inference trees)**.

Application of the inference trees for combined factors revealed 'typical' injured and non-injured groups for horse riding (Figure 2):

1: No jumping performed, intermediate level in horse riding, and one diploma in horse riding.

2: No jumping performed, and a professional level in horse riding.

3: Jumping performed, and no participation in competitions.

4: No jumping performed and no experience in horse riding.

The confidence inference tree is illustrated in Figure 2. Of these variables, jumping had the greatest influence on variability.

Figure 2: Inference tree of risk factor combinations in equestrians.

## Discussion

We identified several factors in our study which were more specific to our patient or our control group in contributing to injuries or protecting against them. Caution is due, however, when classifying the categories determined as risk or protective factors. Those that emerged as statistically significant did so only when seen as single factors and not in the context of the distribution of all other factors in the patient and control groups. Multiple regression analysis was therefore applied. Moreover, a combination of different factors plays a preventive role in riding injuries. We identified one 'typical' group of equestrians susceptible to injuries and three 'typical' groups of riders who are more likely to avoid them.

Equestrian activities are especially dangerous because the rider is unrestrained and is travelling on a largely unpredictable animal capable of speeds of up to 70 kph and of kicking with a force of up to 1 tonne [25]. Most severe and fatal equestrian injuries are of neurological origin [25]. As in most other studies, the most frequent injuries in our survey were to the extremities (upper: 17%; lower: 15%) [15]. Injuries to the head accounted for 24% of our sample, and injuries to the spine for 14%.

The injuries in our study were most frequently cased by falls from horses (65%), followed by kicks (19%) and bites (2%), confirming the findings of Loder RT [15,26].

We found that young women aged 16-30 were the largest group of injured persons and that women accounted for 76% of our sample. This is higher than figures of 42% and 66% reported elsewhere [15,16]. Despite this, multiple regression analysis in our sample showed that being a younger woman or the fact that women are more often involved in equestrian activities than men does not seem to be a reason why more women riders sustain injuries. Further preventive campaigns should target especially this group of young females as we think they might be more susceptible for changes in safer horse riding practice than equestrians already riding for many years.

The type or gender of the horse did not emerge as risk factors for injury in our study. Further studies with larger numbers of different types of horses are warranted to clarify this question.

Other researchers stated that "experience is not protective, helmets are" or "the risk of serious injury seems to be a function of cumulative exposure to horses, not of level of expertise as is assumed by the majority of riders". Our study showed that a higher level of equestrian proficiency and an equestrian diploma were significant factors in avoiding injuries [25,11]. We observed an even lower injury rate for equestrians with more than only one diploma in horse riding. Our results are supported by Newton et al and also by a recent study from the USA by Mayberry et al., who defined the level of equestrian experience according to hours spent with the horse per month and also found that novice riders are at greater risk for injury, and that the incidence of injuries declined with and increasing level of proficiency [27,28]. In the single regression analysis in our survey, spending 20-29 h per week with one's own horse correlated with the lowest injury rate.

Wearing a helmet and a protective waistcoat were significant protective factors in our single regression analysis. Equestrians wearing a helmet also had a 50% lower risk of suffering injury. However, this result implies some limitation in defining the use of helmets as all protective devices were not analyzed and compared specific to certain injuries. The reason why equestrians wearing protective gear had a lower injury rate remains unclear. They might be in general more conscious and less risky characters. Although, multiple regression analysis revealed no significant decrease for the variable "readiness for risk".

The groups of riders less likely to suffer injury could be interpreted as the following:

• Experienced equestrians with a first diploma in horse riding who do not do show jumping and have not suffered previous injuries.

• Experienced and skilled riders who invest several hours a week in horse riding and prefer riding styles other than jumping.

• Less experienced and less ambitious equestrians who perform show jumping once in a while but never take part in competitions.

The group of riders more likely to suffer injury were beginners who did not do show jumping.

### Limitations

Patients and controls were not interviewed consecutively and participation was voluntary. Patients were interviewed in the emergency department after injury and, controls were approached after horse riding. This method of enrolment was chosen for practical reasons and the influence of the different sampling procedures in the study groups cannot be estimated. There is the possibility of greater variability in the patient group leading to wider confidence intervals and more conservative conclusions. After an injury, patients may overestimate or underestimate their readiness for risk and speed leading to recall bias. The extent and effect of this kind of bias cannot be estimated.

Some questions, like readiness for risk and speed, were answered by self-estimation which can also cause information bias. The visual analogue scale (VAS) for the readiness for speed and risk has not been validated and this may limit its value. Nevertheless, VAS is a widely used tool in clinical medicine and provided a good basis for assessments in this study. In general, VAS investigations have been validated for emotions and 'feelings' in the past, and the readiness for speed and to take risk are certainly governed by as feelings [29].

Patients suffering from concussion were only interviewed if their GCS was 15 and they were able to answer the questions fully and coherently. Nevertheless, this subpopulation might be more influenced from recall bias then the rest of the study population.

## Conclusions

Knowledge of risk factors and protective measures is the most important aspect in preventing equestrian injuries. Equestrian injuries are a public health issue, not only for riders, but for everyone in close contact with horses [1,30] Equestrian injury prevention initiatives should further define groups at risk and focus on safe riding practices, proper horse handling, and educating riders in horse behavior. Educational levels and injury risk should be graded and horse riders should be appropriately trained in taking preventative measures. An educational level-injury risk index would be ideal, as for other sports such as golf or paragliding, to track improvement in skills.

By analysing possible risk factors in horse riding we hope to have contributed to expand the evidence base around injury risks in horse riding. Further work should concentrate on systematic reviews of the current literature to suggest guidelines to improve preventive strategies.

## Competing interests

No benefits in any form have been received or will be received from a commercial party related directly or indirectly to the subject of this article.

## Authors' contributions

RMH: Setting up of the questionnaire and writing of the paper

LG: Collection of the data and setting up of the questionnaire

LB: Writing of the paper and data structuring

AS: Methodological advice and performing statistical analysis

HZ: Setting up of the study design and statistical advice

AKE: Setting up of the study design, methodological advice and revision of the manuscript

All authors have read and approved the final manuscript.
